# Effect of Thermo-Sonication and Ultra-High Pressure on the Quality and Phenolic Profile of Mango Juice

**DOI:** 10.3390/foods8080298

**Published:** 2019-07-29

**Authors:** Abdul Ghani Dars, Kai Hu, Qiudou Liu, Aqleem Abbas, Bijun Xie, Zhida Sun

**Affiliations:** 1 College of Food Science and Technology, Huazhong Agricultural University, Wuhan 430070, China; 2 Department of Plant Pathology, College of Plant Science and Technology, Huazhong Agricultural University, Wuhan 430070, China

**Keywords:** mango juice, thermo-sonication, ultra-high pressure, physicochemical property, phenolic compounds

## Abstract

Consumer demand for safe and nutritious fruit juices has led to the development of a number of food processing techniques. To compare the effect of two processing technologies, thermo-sonication (TS) and ultra-high pressure (UHP), on the quality of mango juice, fresh mango juice was treated with TS at 25, 45, 65 and 95 °C for 10 min and UHP at 400 MPa for 10 min. The phenolic profile of mango was also analyzed using the newly developed ultra-performance liquid chromatography-electrospray ionization-quadrupole time of flight-mass spectrometry (UPLC-Q-TOF-HRMS^n^) and, based on this result, the effect of TS and UHP on the phenolics of mango juice was evaluated. Both treatments had minimal effects on the ^o^Brix, pH, and titratable acidity of mango juice. The residual activities of three enzymes (polyphenol oxidase, peroxidase, and pectin methylesterase), antioxidant compounds (vitamin C, Total phenolics, mangiferin derivatives, gallotannins, and quercetin derivatives) and antioxidant activity sharply decreased with the increase in the temperature of the TS treatment. Nevertheless, the UHP treatment retained antioxidants and antioxidant activity at a high level. The UHP process is likely superior to TS in bioactive compounds and antioxidant activity preservation. Therefore, the mango juice products obtained by ultra-high-pressure processing might be more beneficial to health.

## 1. Introduction

Mango *(Mangifera indica* L.) is one of the most economically important and popular tropical fruits and is widely recognized worldwide for its admirable sensorial characteristics (sweet taste, bright color, and delicious flavor) and nutritional composition (carbohydrates such as glucose, fructose and sucrose, vitamins, minerals, fiber, and phytochemical), as well as its popularity and high production. Mango is considered as a ‘king of fruits’ due to extensive appeal across Africa, the Americas, Australia, Europe and Asia [1,2]. 

Mango is consumed as fresh fruit as well as processed products, like juice, pulp, powder, mash, pickles and syrup [2,3] and, among these products, juice has the higher consumption and higher economic value [2,4]. Due to a higher consumption, the mango juice market has increased considerably [5,6]. As for fruit juice processing, a number of studies have focused on the effects of processing technologies on juice quality [2,3,7,8]. Among them, thermal processing was widely applied in the food industries to preserve juice. However, since fruits are usually susceptible to thermal processing, this can lead to considerable damage to bioactive constituents and sensory characteristics of fruit products. Studies on non-thermal processing technologies such as ultraviolet, pulsed electric field, ultrasound and ultra-high pressure (UHP) are being carried out because they cater to the consumer demand of fresh-like, minimally processed foods. When high power ultrasound at low frequencies (20–100 kHz) propagates in liquid, cavitation (formation and collapse of bubbles) occurs. As a result, elevation of localized pressure and temperature causes the alteration of the properties of food products either physically or chemically [7,8]. Individual ultrasound processing effects on juice products were widely reported in different fruit cultivars [8,9,10]. However, few papers about the combinative effects of ultrasound and heat on fruit juice quality are available. UHP is a promising processing technology to meet the needs of fresh juice. At present, the studies are mainly focused on the influence of the technology on macromolecular substances such as protein, pectin and fiber. However, the effect on bioactive components (small molecule) is rarely reported [11]. 

Mango is rich in antioxidants such as polyphenols, flavonoids and other phytochemicals [12]. Previous reports have shown that polyphenols can modulate immune response activities [13,14]. In addition, polyphenols prevent genetic toxicity by reducing the exposure to oxidative and carcinogenic factors [15,16]. Regular consumption of mango fruits supplies a considerable number of polyphenols which have beneficial physiological effects [17,18,19]. Mango polyphenols exhibit anti-inflammatory and cancer cytotoxic properties in multiple cancer types, including malignancies of the colon and breast [13,15,16]. A previous study showed that the content of polyphenols in mango peel is higher than that in pulp [14]. Mango polyphenolics are mainly rich in gallic acid, gallotannins, galloyl glycosides, and flavonoids [12,20]. It has been proved that these phenolic compounds in mango are the main bioactive ingredients beneficial to health [19]. However, no information about the effect of TS and UHP treatments on the phenolic profile of mango juice is available. Current consumers look for healthy food products, maintaining mango juice’s inherent physical and chemical properties of interest (e.g., texture, pH, titratable acidity, total soluble solids, total phenolics, total carotenoids and vitamin C) and nutritional quality during its processing. Hence, the objective of the present study was to evaluate the effects of TS and UHP treatments on the quality and phenolic profile of mango juice.

## 2. Materials and Methods

### 2.1. Preparation of Juice Samples

Chinese mangoes cv. Kensington Pride were purchased from a producer in Hainan province and brought to full ripeness by maintaining at 20–23 °C and 90% relative humidity (RH). Mangoes were washed with tap water, then dried and cut into small pieces. Kernel and bruised portions were discarded. Peel and a small part of pulp were collected and stored at −20 °C for phenolic identification within two weeks. The remaining pulp was used to obtain juice by a domestic juice extractor (AUX-PB953, Foshan Haixun Electric Appliances Co., Ltd., Foshan, China). After filtration using a sterilized double-layered muslin cloth, the juice was vortex mixed and stored in 50-mL pre-sterilized PET bottles at 4 °C for further treatment within 2 h. 

### 2.2. Thermo-Sonication (TS) and Ultra-High Pressure (UHP) Treatment

TS treatment was performed using a 250-W ultrasonic processor (Ningbo Xingzhi Biotechnoligy Co., Ltd., Ningbo, China) at four different temperatures 25, 45, 65, and 95 °C for 10 min. UHP treatment was carried out using an ultra-high pressure processor (Bao Tou KeFa High Pressure Technology Co., Ltd., Baotou, China) at 400 MPa for 10 min (based on previous optimization). The juice without treatment was considered as control. All treatments were conducted in triplicate. Brix, pH, acidity, polyphenol oxidase (PPO), peroxidase (POD), and pectin methylesterase (PME) were measured immediately after treatment. The remaining juice was stored at −20 °C until further analysis for vitamin C, total phenolic content, antioxidant activity and quantification of phenolic compounds within two weeks.

### 2.3. Determination of ^o^Brix, pH, and Acidity 

A hand refractometer WYT-80 (Quanzhou Wander Experimental Instrument Co., Ltd., Quanzhou, China) was used to measure ^o^Brix. A digital pH meter (Delta 320 pH meter, Metller Toledo Instruments Co., Ltd., Shanghai, China) was used to measure pH. The titratable acidity was measured according to the method suggested by the “Association of Official Analytical Chemists” (AOAC, 2000) with a 0.1 M NaOH solution as the titration solution.

### 2.4. Determination of PPO, POD and PME Residual Activities

The samples of mango juice were centrifuged at 8000 rpm for 15 min at 4 °C for the enzyme activity assay. Polyphenol oxidase (PPO) and peroxidase (POD) activity were measured according to the protocol of Macdonald and Schanchke [21]. For the determination of PPO, 1.5 mL supernatant was mixed with 0.5 mL catechol (0.5 mol/L) and 3.0 mL potassium phosphate buffer (0.2 mol/L, pH 6.8). The absorbance was recorded at 410 nm within 3 min. With respect to POD, 0.32 mL potassium phosphate buffer (0.2 mol/L, pH 6.8), 0.32 mL pyrogallol (5 g/100 mL) and 0.6 mL H_2_O_2_ (0.147 mol/L) were mixed and variation in absorbance at 420 nm within 3 min was noted. Pectinmethylesterase (PME) assay was conducted according to the method used by Saeeduddin et al. [7]. Briefly, 10 mL supernatant was mixed with 40 mL pectin solution (1 g/100 mL) containing 0.15 mol/L NaCl. The pH was adjusted to 7.7 by the addition of 0.05 mol/L NaOH and the time taken was recorded. The reaction system was incubated at 50 ± 2 °C. Residual activities of PPO, POD and PME were calculated using the following equation:

Enzyme activity (%) = 100A_t_/A_0_ (1)

where A_0_ and A_t_ are the enzyme activities of the control and treatment samples respectively. 

### 2.5. Determination of Total Phenolic Content 

The total phenolic content was determined according to the method carried out by Tong et al. [22]. Approximately 100 μL mango juice from each sample was mixed with 0.4 mL distilled water and 0.5 mL diluted Folin–Ciocalteu reagent (1:10, v:v). The mixtures were incubated for 5 min at room temperature and 1 mL 7.5% sodium carbonate (*w*/*v*) was added. The absorbance was measured at 765 nm after maintaining at 30 min in dark. A standard curve was obtained with gallic acid and the result was expressed as mg of gallic acid equivalents (GAE)/mL juice. 

### 2.6. Determination of Vitamin C

Vitamin C content was determined using a simplified method reported by Sulaiman and Ooi [23]. Approximately 25 mL of diluted solution was titrated against 0.1‰ 2, 6-dichlorophenolindophenol sodium (DCIPS) until the solution became a light pink color and persisted for 15 s. The calibration of 0.1‰ DCIPS solution was performed with 1 mg/mL ascorbic acid. The results were calculated and expressed as mgL^−1^ juice.

### 2.7. Determination of Total Antioxidant Activity

The total antioxidant activity assay was tested using the method reported by Li et al. [24] The juice (0.4 mL) was centrifuged and mixed with 4 mL reagent mixture (sulfuric acid (0.6 mol/L), sodium phosphate (28 mmol/L) and ammonium molybdate (4 mmol/L)). The mixture was kept at 95 °C for 90 min and the absorbance was measured at 695 nm. Ascorbic acid was used as standard and the result was presented as mg ascorbic acid equivalent/mL juice.

### 2.8. Extraction of Polyphenolic Compounds for UPLC/UPLC-Q-TOF-HRMS^n^

Mango pulp was thawed and homogenized in an appropriate ratio of 10 g of pulp to 30 mL of a solvent mixture (ethanol/methanol/acetone, 1/1/1). Similarly, mango peel was thawed and homogenized in a ratio of 5 g of peel to 15 mL of a solvent mixture (ethanol/methanol/acetone, 1/1/1). For the quantification analysis of polyphenolics in mango juice before and after different treatments, the extraction was conducted using the same method used for mango pulp. Afterwards, the resultant solution of pulp, peel and juice was filtered through cheese cloth. The solvents were removed at 40 °C by rotary evaporation under reduced pressure, and the aqueous residue was centrifuged to remove insoluble precipitates. Polyphenolics were partitioned in a 20-mL Waters C18 cartridge. Compounds not adsorbed to the cartridge were partitioned into ethyl acetate using a separatory funnel. The ethyl acetate phase was combined with the methanol elute from the C18 cartridges, and the solvent were removed under reduced pressure. The residual was dissolved in chromatographic acetonitrile and used for UPLC and UPLC-Q-TOF-HRMS^n^.

### 2.9. The Quantification and Identification of Polyphenolic Compounds in Mango by UPLC/UPLC-Q-TOF-HRMS^n^

For the quantification and identification of polyphenolic compounds, LC analysis was conducted using Acquity Ultra Performance Liquid Chromatography system (Waters, Milford, MA, USA), equipped with a C18 column (2.1 × 100 mm, 1.7 μm, Waters, Milford, MA, USA). The column, constant at 40 °C, was eluted with a linear gradient mobile phase at 0–28 min: 2–50% B, 28–28.5 min: 50–100% B, 28.5–30.5 min: 100% B, 30.5–32 min: 100–2% B, 32–34 min: 2% B, where A = water with 0.1% acetic acid and B = acetonitrile. The flow rate was 0.3 mL/min, and the injected volume was 1 μL. 

The mass spectrometric data of the full scan mode was collected using a G2-XS QT of MS (Waters, Milford, MA, USA). The scan range was from *m*/*z* 100 to 2000 with a scan time of 0.3 s. The source temperature was set at 120 °C with a cone gas flow of 50 L/h. The gas flow was set to 800 L/h at a temperature of 400 °C. The capillary was set at 1 kV for ESI^−^ mode with the cone voltage at 40 V. The MS^n^ analysis was carried out on a Waters-Micro mass Quattro Premier triple quadrupole mass spectrometer. The collision energy was optimized according to the specific precursor ions. 

### 2.10. Statistical Analysis

All tests were performed in triplicate and data were expressed as the means ± the standard deviation. One-way analysis of variance (ANOVA) followed by LSD multiple comparison were conducted using the SPSS 20 (IBM, Armonk, NY, USA). Differences with a *p* value < 0.05 were considered significant. 

## 3. Results and Discussion

### 3.1. Effects of TS and UHP on ^o^Brix, pH and Titratable Acidity

The effects of TS and UHP on ^o^Brix, pH and titratable acidity are shown in Table 1. All treatments were not significantly different from each other when addressing the ^o^Brix, pH and titratable acidity of mango juice. These results are in consistent with the previous reports [9,10,25]. These previous studies showed no significant variations in the ^o^Brix, pH and titratable acidity of other fruit juices as a result of various non-thermal processing food technology. This indicated that TS and UHP are promising tools since they improved the quality of fruit juice without causing the significant change of basic physicochemical indexes. 

### 3.2. Effects of TS and UHP on Inactivation of PPO, POD and PME

The effects of different TS treatments on PPO, POD and PME in mango juice are shown in Table 2. The highest enzyme inactivation was exhibited in the sample treated with TS at 95 °C, which showed the residual activities of PPO, POD and PME as 3.47, 1.61 and 2.24% respectively. The increase in temperature interval significantly deactivates PPO, POD and PME as described in previous report [26]. The time duration of the ultrasonic treatment causes the formation of free radicals, which are then involved in inactivation of enzyme activities [27]. The formation of cavities due to bubbles development and disappearance is related to enzyme inactivation [28]. This can induce sharp increase in temperature and pressure in a localized ultrasound generating area, which may be a major factor in enzyme deactivation. As a result, the temperature and other mechanical forces during ultrasonic pasteurization have a combined role in the enzyme inactivation. The mango juice was treated with UHP treatment at 400 MPa for 10 min. However, just a slight effect on the enzyme inactivation was recorded. These three enzymes were likely pressure resistant, and the corresponding mechanism needs to be further studied.

### 3.3. Effects of TS and UHP on Antioxidant Compounds and Antioxidant Capacity

The effects of thermo-sonication on ascorbic acids and total phenolics are mentioned in Table 3. The amount of ascorbic acid in fresh mango juice (control) was 117.47 ± 1.12 mg/L. It sharply decreased from 116.26 ± 0.89 to 33.12 ± 1.35 mg/mL with the temperature increasing from 25 to 95 °C, corresponding to 98.97, 64.22, 47.16 and 28.19% of residual quantity when TS treatments were conducted at 25, 45, 65 and 95 °C, respectively. There is no significant difference between the vitamin C contents of control and TS at 25 °C. This means TS at normal temperature has no effect on the vitamin C contents of mango juice. However, it exerts a significant effect of decomposition or oxidation of vitamin C when the temperature is high (>45 °C) and, with the increase in temperature, the loss of vitamin C becomes more prominent. Similar results were reported in apple juice and watermelon juice when treated with TS at different temperatures [29,30]. From Table 3, vitamin C content was 114.16 ± 1.02 mg/mL when treated with UHP at 400 MPa, showing a slight reduction (2.82%) compared with control. It is likely that the density of the reactive system increased during UHP treatment, which promoted the decomposition of vitamin C. The total phenolic content of the control sample was 1.76 ± 0.08 mg/mL. It decreased from 1.73 ± 0.05 to 0.592 ± 0.005 mg GAE/mL, with the temperature increasing from 25 to 95 °C, reducing by 1.70, 51.36, 58.86 and 66.36% respectively. This suggests that TS at relatively low temperature has no significant effect on total phenolic content of mango juice, but significantly reduce it when temperature is high. A similar result was obtained in pear juice [7]. Mango juice treated with UHP at 400 MPa showed a higher total phenolic content (1.82 ± 0.003) than the control, though this is not significant. The increase in the content of phenolic compounds during UHP treatment might be due to the secretion of the bound forms of these compounds in juice [4,31]. At 25 °C, vitamin C and polyphenol content hardly changed after ultrasonic treatment as compared to control. This indicated sonication only exerted a minor effect on antioxidants. Nevertheless, the high reduction in the content of ascorbic acid and total phenols at 45–95 °C indicates that both entities were highly heat sensitive. UHP is a promising non-thermal technology for food processing and can effectively protect bioactive components and avoid losses caused by heat treatment. 

The effects of TS on the antioxidant capacity of mango juice are shown in Table 3. There was no significant difference in total antioxidant activity between control (0.867 ± 0.006 mg AAE/mL) and TS25 (0.862 ± 0.008 mg AAE/mL) samples. However, with the increase in temperature from 45 °C to 95 °C, the total antioxidant activity decreased from 0.792 ± 0.006 to 0.572 ± 0.005 mg AAE/mL, leading to 8.65–34.03% loss. The antioxidant capacity of fruit juice is attributed to the presence of antioxidant compounds such as ascorbic acid and total phenol [9]. The decreasing trend of the antioxidant capacity of samples is consistent with the decrease in vitamin C and polyphenol content by TS treatments at high temperature. However, ultra-high-pressure treatment was found to retain the antioxidant activity (0.831 ± 0.003 mg AAE/mL) of mango juice at a high level of 95.85%, which indicates that UHP is a promising technique for the protection of antioxidant and free radical scavenging capacity.

### 3.4. Identification of Polyphenolic Compounds

The polyphenols in mango pulp and peel were identified by UPLC-Q-TOF-HRMS^n^ (Supporting Information, Figures S1 and S2), and the results are shown in Table 4 and Table 5 respectively. Approximately 22 compounds from the peel and 14 compounds from the pulp were identified using the UPLC-Q-TOF-HRMS^n^ technique. All of the phenolic compounds identified in mango pulp, except for iriflophenone di-*O*-galloyl-glucoside, were included in those of peel. Therefore, the MSs of phenolic compounds in mango peel are discussed here (Table 4).

Among these compounds, five compounds, i.e., **2**, **4**, **9**, **10** and **11**, were identified as benzophenone derivatives (Table 4). The molecule ions at m/z 575.1039, 727.1071 and 879.1256 were identified as maclurin mono-*O*-galloyl-glucoside (compound **2**), maclurin-di-*O*-galloyl-glucoside (Compounds **4**, **10** and **11**) and maclurin tri-*O*-galloyl-glucoside (Compound **9**). The fragment ions were obtained by the successive loss of galloyl or H_2_O. The MS ^2^ results showed that the fragment ions of compound **2** were found at *m*/*z* 423.09, 303.05, 285.04, 261.04 and 193.02, corresponding to the loss of galloy moiety, 272.0469 Da ion, H_2_O, 2H_2_O and 110.0352 Da ion, respectively. Similar fragment loss was found in compounds **4**, **9**, **10** and **11**. The above results show that benzophenone derivatives in mango were all maclurin-gallic glucosides with different substitution degrees. Compounds **3**, **6** and **7** were identified as mangiferin and their derivatives (Table 4). Compound **3** was identified and detected as mangiferin at *m*/*z* 421.0771 ([M − H]^−^). Mangiferin is a glycosylated xanthine found in several varieties of mango. It is reported to be not only a typical biomarker for resistance against *Fm* infection but also have pharmacological activities in different organs and tissues, such as protecting the heart, neurons, liver, and kidneys and preventing or delaying the onset of diseases [32]. Compounds **6** and **7** were identified as mangiferin gallate and iso-mangiferin gallate as a result of the loss of galloyl moiety and H_2_O. In this study, five tannins, compounds **1**, **5**, **8**, **16** and **20**, were identified as gallic acid, tetra-*O*-galloyl-glucoside, iso-tetra-*O*-galloyl-glucoside, penta-*O*-galloyl-glucose and hexa-*O*-galloyl-glucose, respectively. Gallic acid is a widespread tannin in mangoes and has been recognized in other cultivars of mangoes [32]. In common, for most ions, neutral losses of the galloyl fraction (152 Da) and of gallic acid (170 Da) were shown in Table 4. The fragmentation profile created for these polyphenolic compounds were related to gallotannins and benzophenone derivatives [12]. Compounds **12**, **13**, **14**, **15**, **17**, **18**, **19**, **21** and **22** were found to be flavonoids and identified as quercetin 3-*O*-galactoside, quercetin 3-*O*-glucoside, iso-quercetin 3-*O*-glucoside, quercetin 3-*O*-xyloside, iso-quercetin 3-*O*-glucoside, iso-quercetin 3-*O*-xyloside, kaempferol 3-*O*-glucoside, quercetin 3-*O*-rhamnoside and iso-quercetin 3-*O*-rhamnoside, respectiviely. These flavonoids were mainly of different forms of glycosides. Among them, the quercetin compound was the major aglycone. The phenolic compounds identified in pulp are quite similar to the compounds of peel. However, one compound in pulp was different from peel. This compound was identified as iriflophenone di-*O*-galloyl-glucoside at *m*/*z* 711.1124 ([M − H]^−^). Specific fragmentation patterns of all the identified compounds are shown in the Supplementary Materials. 

### 3.5. Effects of TS and UHP on Phenolic Groups

**Figure 1:**
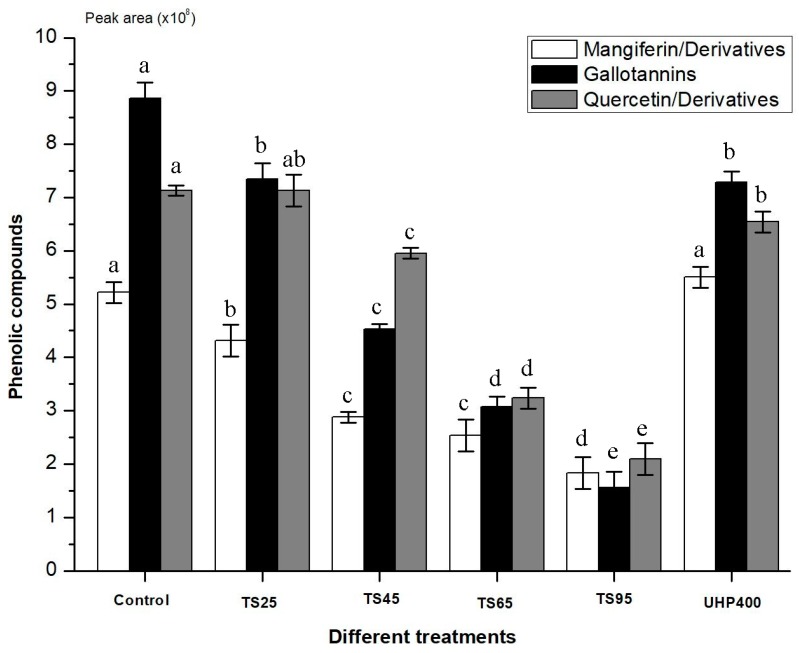
Effects of thermo-sonication (TS) and ultra-high pressure (UHP) on phenolic groups, mangiferin and its derivatives, quercetin derivatives and gallotannins. TS25, TS45, TS65 and TS95 represent thermo-sonication treatment at 25, 45, 65 and 95 °C. UHP400 represents ultra-high pressure at 400 MPa for 10 min. Each column represents a mean and the vertical bars indicate the standard deviation. Means with different letters within the same color columns are significantly different (LSD test, *p* < 0.05).

Based on the analysis of UPLC/UPLC-Q-TOF-HRMS^n^, as well as the distribution and structure characteristics of phenolic compounds in the chromatogram, the phenolic compounds in mango juice were divided into three main groups, (i) mangiferin/derivatives, (ii) gallotannins, and (iii) quercetin derivatives. Each group was relatively quantified by peak area to study its variation during TS and UHP treatments. As shown in Figure 1, all three phenolic groups exhibited a generally decreasing trend with the development of temperature, which was in accordance with the changes of total phenolics. When treated with TS at 95 °C, the content of phenolic groups in mango juice was lowest. However, quercetin derivatives showed an ultrasonic-resistant ability at low treated temperature (25 °C), but other two kinds of phenolic compounds were unstable under ultrasound treatment, likely due to oxidation and degradation induced by the ultrasonic cavitation effect. In addition, all three phenolic groups were temperature-sensitive. A previous study showed that thermal processing significantly (*p* < 0.05) affected individual phenolic acids, anthocyanins, flavan-3-ols, and flavonols, significantly (*p* < 0.05) reduced total phenolic acid contents in both pinto and black beans and total flavonol contents in pinto beans, and dramatically reduced anthocyanin contents in black beans [33]. The flavonols rutin and quercetin also degraded under thermal processing in an aqueous model system [34]. The combined mangiferin/derivatives may be released by UHP. This shows that ultra-high pressure could be an excellent process technology of mango juice, with a high retention rate of phenolic groups (Figure 1).

## 4. Conclusions

TS and UHP had a minor effect on the basic physicochemical properties of mango juice. TS at high temperature significantly reduced the enzyme activities of PPO, POD and PME. The effects of TS treatment on vitamin C, total phenolics and antioxidant activity were significant, and mainly degradative or oxidative action. However, UHP treatment gave a high level of antioxidants and antioxidant activity of mango juice. The Kensington Pride mango researched in this study was cultivated in Hainan, the southernmost province of China. Abundant phenolic compounds from mango were identified and the effects of TS and UHP on the phenolic profile were analyzed. We find that TS induced the significant degradation of phenolic groups, mangiferin/derivatives, gallotannins, and quercetin derivatives. The UHP treatment was likely superior to TS in bioactive compounds and antioxidant activity preservation except for browning-related enzymes, which needs further study. The mango juice products obtained by ultra-high-pressure processing are more beneficial to health. 

## Figures and Tables

**Table 1:** Effect of different treatments on Brix, pH, titratable acidity in mango juice *^a^*.

Treatments *^b^*	^o^Brix	pH	Titratable Acidity (%)
Control	11.80 ± 0.02a	4.75 ± 0.03a	0.16 ± 0.01a
TS25	11.80 ± 0.01a	4.74 ± 0.03a	0.16 ± 0.01a
TS45	11.72 ± 0.02a	4.73 ± 0.01ab	0.17 ± 0.01a
TS65	11.60 ± 0.03a	4.69 ± 0.02b	0.17 ± 0.01a
TS95	11.54 ± 0.06a	4.67 ± 0.02bc	0.17 ± 0.01a
UHP400	11.62 ± 0.04a	4.70 ± 0.02c	0.17 ± 0.01a

*^a^* Data are presented as means ± the standard deviation. Means with different letters within a column are significantly different (LSD test) at *p* < 0.05. *^b^* TS25, TS45, TS65 and TS95 represent thermo-sonication treatment at 25, 45, 65 and 95 °C. UHP400 represents ultra-high pressure at 400 MPa for 10 min.

**Table 2:** Effect of different treatments on the residual activity percentage of POD, PPO and PME in mango juice *^a^*.

Treatment *^b^*	POD (Residual Activity %)	PPO (Residual Activity %)	PME (Residual Activity %)
Control	100.00 ± 00a	100.00 ± 00a	100.00 ± 00a
TS25	92.57 ± 0.94c	87.73 ± 1.30c	90.76 ± 1.82c
TS45	51.42 ± 1.22d	45.44 ± 2.11d	48.36 ± 1.10d
TS65	37.45 ± 1.15e	31.39 ± 1.71e	34.52 ± 0.77e
TS95	3.47 ± 0.68f	1.61 ± 0.57f	2.24 ± 0.57f
UHP400	98.18 ± 0.80b	93.26 ± 0.82b	96.46 ± 1.76b

*^a^* Data are presented as means ± the standard deviation. Means with different letters within a column are significantly different (LSD test) at *p* < 0.05. *^b^* TS25, TS45, TS65 and TS95 represent thermo-sonication treatment at 25, 45, 65 and 95 °C. UHP400 represents ultra-high pressure at 400 MPa for 10 min.

**Table 3:** Effect of different treatments on antioxidants compounds and antioxidant activity *^a^*.

Treatment *^b^*	Vitamin C (mg/L)	Total Phenolic Content (mg GAE/mL) *^c^*	Total Antioxidant Capacity (mg AAE/mL) *^d^*
Control	117.47 ± 1.12a	1.76 ± 0.08ab	0.867 ± 0.006a
TS25	116.26 ± 0.89ab	1.73 ± 0.05b	0.862 ± 0.008a
TS45	75.45 ± 1.04c	0.856 ± 0.006c	0.792 ± 0.004c
TS65	55.40 ± 0.71d	0.724 ± 0.008d	0.716 ± 0.008d
TS95	33.12 ± 1.35e	0.592 ± 0.005e	0.572 ± 0.005e
UHP400	114.16 ± 1.02b	1.82 ± 0.003a	0.831 ± 0.003b

*^a^* Data are presented as means ± the standard deviation. Means with different letters within a column are significantly different at (LSD test) *p* < 0.05. *^b^* TS25, TS45, TS65 and TS95 represent thermo-sonication treatment at 25, 45, 65 and 95 °C. UHP400 represents ultra-high pressure at 400 MPa for 10 min. *^c^* Total phenolic content expressed as mg gallic acid equivalent per mL juice. *^d^* Total antioxidant capacity expressed as mg ascorbic acid equivalent per mL juice.

**Table 4:** Identification of polyphenolic compounds in Kensington Pride mango peel by UPLC-Q-TOF-HRMS^n^.

Compound	Retention Time (min)	Identity	Formula	Calculated [M − H] *m*/*z*	Observed [M − H] *m*/*z*	Error (ppm)	Ion Fragment
**1**	1.82	Gallic acid	C_7_H_6_O_5_	169.0137	169.0218	47.93	125.0325, 97.0374
**2**	6.822	maclurin mono-*O*-galloyl-glucoside	C_26_H_24_O_15_	575.1037	575.1039	0.35	303.057, 285.0470, 261.0472, 423.0966, 193.0218
**3**	8.22	mangiferin	C_19_H_18_O_11_	421.0771	421.0814	10.21	
**4**	8.283	maclurin di-*O*-galloyl-glucoside	C_33_H_28_O_19_	727.1147	727.1071	−10.45	
**5**	8.956	tetra-*O*-galloyl-glucose	C_34_H_28_O_22_	787.0994	787.0882	−14.23	635.0850, 617.0764
**6**	9.567	mangiferin gallate	C_26_H_22_O_15_	573.088	573.0867	−2.27	403.0731
**7**	10.009	iso-mangiferin gallate	C_26_H_22_O_15_	573.088	573.0974	16.40	421.0774
**8**	10.177	iso-tetra-*O*-galloyl-glucose	C_34_H_28_O_22_	787.0994	787.0882	−14.23	635.0854, 617.0757
**9**	10.33	maclurin tri-*O*-galloyl-glucoside	C_40_H_32_O_23_	879.1256	879.1214	−4.78	727.1166
**10**	10.477	maclurin di-*O*-galloyl-glucoside	C_33_H_28_O_19_	727.1147	727.1071	−10.45	421.0774, 403.0706
**11**	10.682	iso-maclurin di-*O*-galloyl-glucoside	C_33_H_28_O_19_	727.1147	727.1071	−10.45	421.0774, 403.0706
**12**	10.78	quercetin 3-*O*-galactoside	C_21_H_20_O_12_	463.0877	463.0908	6.69	301.0396, 300.0330,
**13**	11.03	quercetin 3-*O*-glucoside	C_21_H_20_O_12_	463.0877	463.0909	6.91	301.0396, 300.0330
**14**	11.439	iso-quercetin 3-*O*-glucoside	C_21_H_20_O_12_	463.0877	463.0909	6.91	301.0396, 300.0330
**15**	11.624	quercetin 3-*O*-xyloside	C_20_H_18_O_11_	433.0771	433.0811	9.24	301.0396, 300.0330
**16**	11.676	penta-*O*-galloyl-glucose	C_41_H_32_O_26_	939.1104	939.101	−10.01	787.0900, 769.0813, 617.0764
**17**	11.906	iso-quercetin 3-*O*-glucoside	C_21_H_20_O_12_	463.0877	463.0909	6.91	301.0397, 300.0333
**18**	12.081	quercetin 3-*O*-xyloside	C_20_H_18_O_11_	433.0771	433.0811	9.24	301.0396, 300.0330
**19**	12.196	kaempferol 3-*O*-glucoside	C_21_H_20_O_11_	447.0927	447.0963	8.05	285.0455, 284.0391, 255.0366
**20**	12.206	hexa-*O*-galloyl-glucose	C_48_H_36_O_30_	1091.1213	1091.1174	−3.57	939.0916
**21**	12.217	quercetin 3-*O*-rhamnoside	C_21_H_20_O_11_	447.0927	447.096	7.38	300.0331
**22**	12.318	iso-quercetin 3-*O*-rhamnoside	C_21_H_20_O_11_	447.0927	447.096	7.38	300.034

**Table 5:** Identification of polyphenolic compounds in Kensington Pride mango pulp by UPLC-Q-TOF-HRMS^n^.

Compound	Retention Time (min)	Identity	Formula	Calculated [M−H] *m*/*z*	Observed [M−H] *m*/*z*	Error (ppm)	Ion Fragment
**1**	1.811	Gallic acid	C_7_H_6_O_5_	169.0137	169.0221	49.70	125.0325
**2**	6.791	maclurin mono-*O*-galloyl-glucoside	C_26_H_24_O_15_	575.1037	575.103	−1.22	303.062
**3**	8.321	maclurin di-*O*-galloyl-glucoside	C_33_H_28_O_19_	727.1147	727.1057	−12.38	
**4**	9.587	mangiferin gallate	C_26_H_22_O_15_	573.088	573.088	0.00	301.0057
**5**	9.783	iriflophenone di-*O*-galloyl-glucoside	C_33_H_28_O_18_	711.1197	711.1124	−10.27	
**6**	10.187	tetra-*O*-galloyl-glucose	C_34_H_28_O_22_	727.1147	787.0897	−12.32	635.0834, 617.0766
**7**	10.445	tetra-*O*-galloyl-glucose	C_34_H_28_O_22_	727.1147	787.0897	−12.32	635.0834, 617.0766
**8**	10.787	quercetin 3-*O*-galactoside	C_21_H_20_O_12_	463.0877	463.0895	3.89	300.032, 301.0393
**9**	11.055	quercetin 3-*O*-glucoside	C_21_H_20_O_12_	463.0877	463.0902	5.40	300.032, 301.0393
**10**	11.439	quercetin 3-*O*-arabinopyranoside	C_20_H_18_O_11_	433.0771	433.0796	5.77	300.0334
**11**	11.76	quercetin 3-*O*-rhamnoside	C_21_H_20_O_11_	447.0927	447.0951	5.37	301.0034
**12**	12.217	kaempferol 3-*O*-glucoside	C_21_H_20_O_11_	447.0927	447.0954	6.04	284.0297, 255.0339
**13**	14.215	penta-*O*-galloyl-glucose	C_41_H_32_O_26_	939.1104	939.1005	−10.54	769.0818
**14**	16.858	maclurin tri-*O*-galloyl-glucoside	C_40_H_32_O_23_	879.1256	879.1292	4.09	

## Supplementary Materials

The following are available online at https://www.mdpi.com/2304-8158/8/8/298/s1, Figure S1: Identification of polyphenolic compounds in Kensington Pride mango peel by UPLC-Q-TOF-HRMS^n^, Figure S2: Identification of polyphenolic compounds in Kensington Pride mango pulp by UPLC-Q-TOF-HRMS^n^.
