# Supplementary material for: Effect of Thermo-Sonication and Ultra-High Pressure on the Quality and Phenolic Profile of Mango Juice
Source: Foods. 2019 Jul 29;8(8):298. doi: 10.3390/foods8080298 (PMC6723886; doi:10.3390/foods8080298)
Supplement: Supplementary file 1 [file foods-08-00298-s001.zip › foods-538587-supplementary.docx]

**supplementary materials**


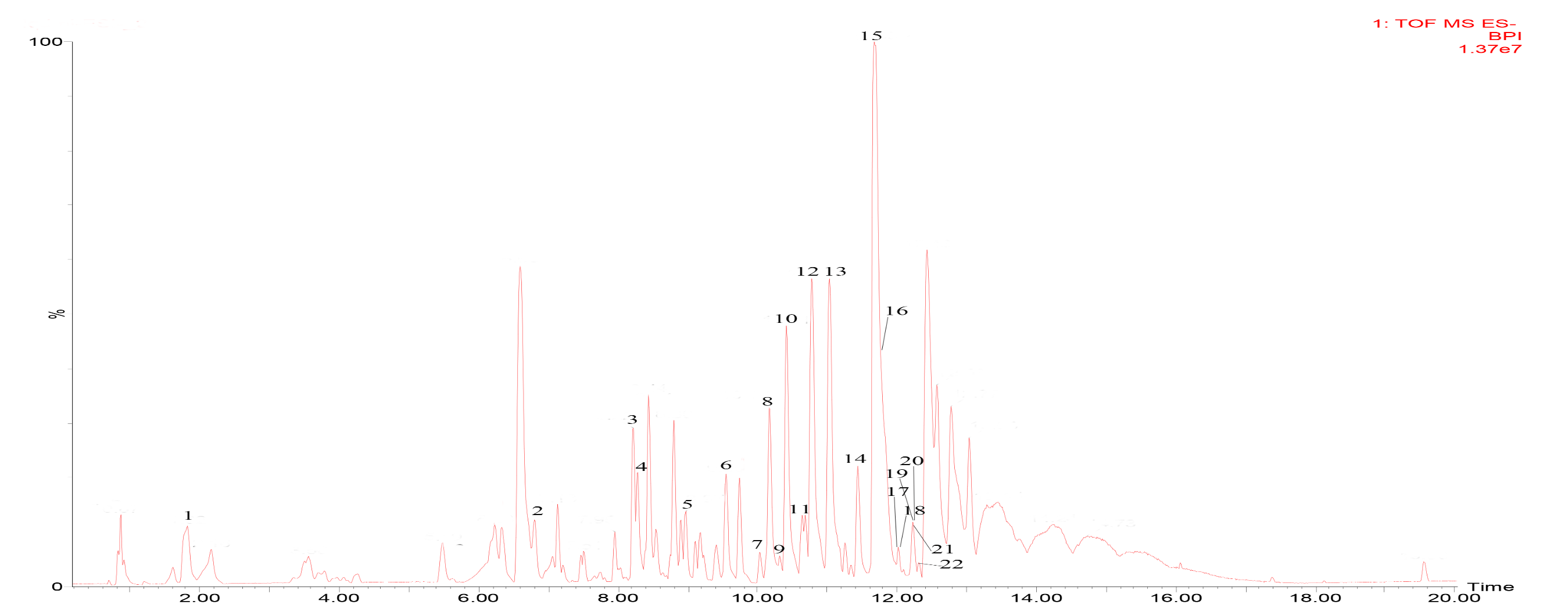


**Supplemental Material, Figure S1.** Identification of polyphenolic compounds in Kensington Pride mango peel by UPLC-Q-TOF-HRMS^n.^


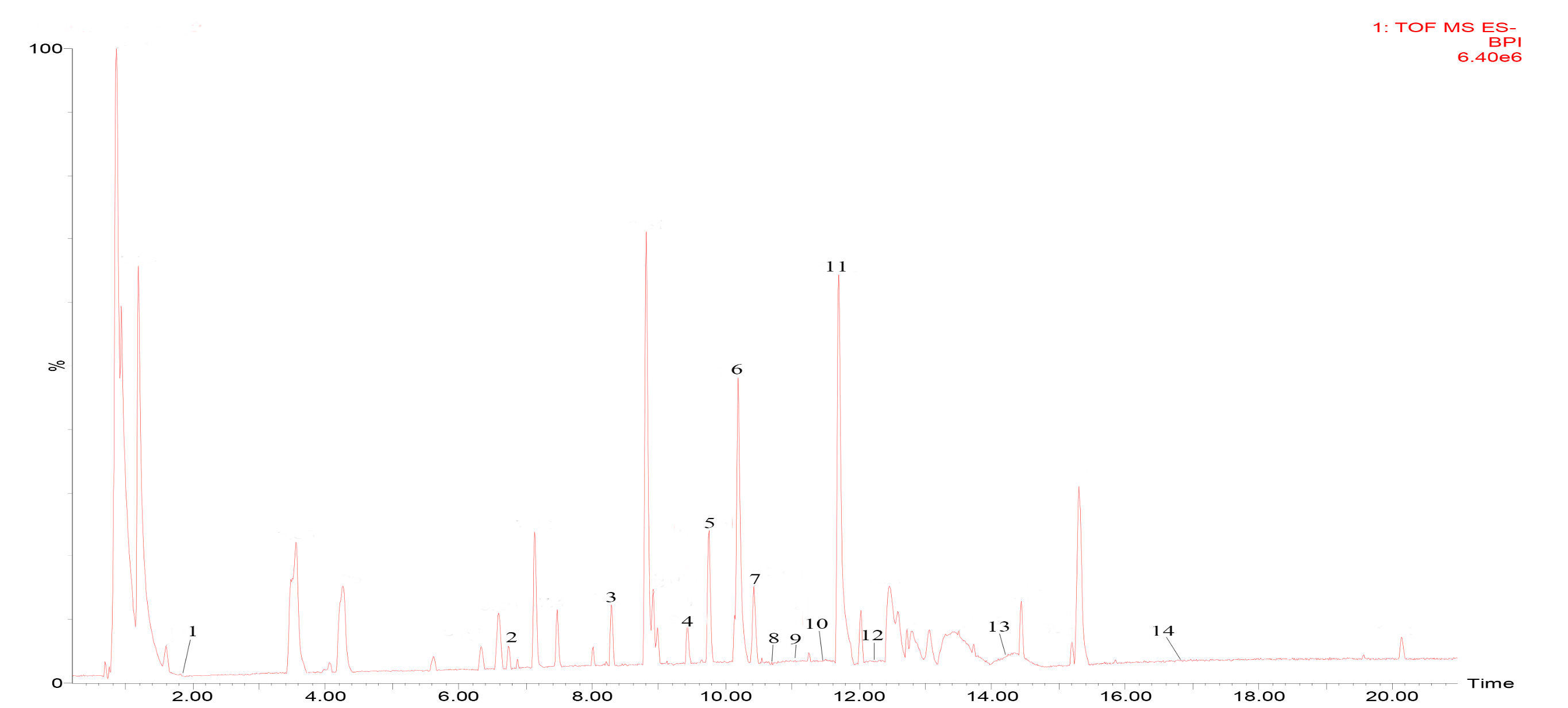


**Supplemental Material, Figure S2.** Identification of polyphenolic compounds in Kensington Pride mango pulp by UPLC-Q-TOF-HRMS^n.^

1. Benzophenone Derivatives

Among these compounds, three compounds i.e. Compound 2, 4, 9, 10 and 11 were identified as Benzophenone derivatives (Table 4). Compounds were detected and the molecule ions at m/z 575.1039, 727.1071 and 879.1256 were identified as maclurin mono-*O*-galloyl-glucoside (compound 2), maclurin-di-*O*-galloyl-glucoside (Compound 4, 10 and 11) and maclurin tri-*O*-galloyl-glucoside (Compound 9). The MS ^2^ results showed that the fragment ions of compound 2 were found at m/z 423.09, 303.05, 285.04, 261.04 and 193.02, respectively. A loss of 272.0469 Da ion in the MS^2^ experiment resulted in the formation of a section ion of m/z 303.0570. The fragment ion (m/z 423.0966) equivalent to the loss of a galloyl moiety (152.0073 Da) was also detected. The MS^2^ experiment yielded a fragment of m/z 193.0218 (303.0570 Da ion loss of 110.0352 Da) as the predominant ion which decarboxylated (loss of 44 Da) in the MS^n^ experiment. The fragment ions at m/z 285.0470 and 261.0472 were 303.0570Da ion lost by one and two water molecules, respectively. However the molecular ion of compound 9 produced MS^2^ fragment ions at *m/z* 727.1166, 421.0774 and 403.0706 which corresponded to the loss of a galloyl moiety (152.0090 Da), 2 galloyl and 2 galloyl + H_2_O. The above results showed that benzophenone derivatives in mango were all maclurin- gallic glucosides with different substitution degrees.

2. Mangiferin and Derivatives

Among the different compounds identified in the peel of mango cv. Ao, compounds 3, 6 and 7 were identified as mangiferin and their derivatives (Table 4,). Compound 3 was identified and detected as mangiferin at m/z 421.0771 ([M-H]-). Mangiferin is a glycosylated xanthine found in several varieties of mango. It is reported to be not only a typical biomarker for resistance against *Fm* infection but also have pharmacological activities in different organs and tissues, such as protecting the heart, neurons, liver, and kidneys and preventing or delaying the onset of diseases[17]. Compound 6 was identified and detected as mangiferin gallate at *m/z* 573.0867 [M-H]^-^ with MS^2^ yielding major fragment at *m/z* 403.0731. Similarly compound 7 was identified and detected iso-mangiferin gallate at m/z 573.0974 with major fragment at m/z 421.0774. The fragment ion at m/z 421.0774 was ([M-H]^-^) at *m/z* 573.0974 that loses a galloyl moiety and H_2_O.

3. Gallates and Gallotannins

In this study, five tannins, gallic acid, tetra-*O*-galloyl-glucoside, iso-tetra-*O*-galloyl-glucoside, penta-*O*-galloyl-glucose and hexa-*O*-galloyl-glucose were identified. Gallic acid (compound 1) is widespread tannin in mangoes and has been recognized in other cultivars of mangoes [17]. In common, for most ions, neutral losses of the galloyl fraction (152 Da) and of gallic acid (170 Da) were shown in Table 4. The fragmentation profile created for these polyphenolic compounds were related to gallotannins and benzophenone derivatives [4]. Compound 1 was detected and identified as gallic acid at m/z 169.0218 [M-H]^-^ and fragment ions was identified at m/z 125.0325 (M-H-CO2).  Tetra-*O*-galloyl-glucoside (Compound 5) showed a quasi-molecular ion ([M-H]^-^) at *m/z* 787.0882 compared with theoretical value, and yielded characteristic ions at m/z 635.0851 (617.0760-H_2_O) and 617.0764 (787.0882-C_6_O_5_H_10_+H) affected by the loss of galloyl (152 Da) and gallic acid (170 Da) moieties, respectively. Similarly iso- tetra-O-galloyl-glucose (Compounds 8) was identified and detected at *m/z* 787.0994 ([M-H]^-^) compared with theoretical value. However it was found to have similar postulated fragmentation pathway to compound 5. Compound 16 gave a [M - H]^-^ ion *m/z* 939.1010 (error, -10.01 ppm) and fragment ions at *m/z* 787.0900, 769.0813, 617.0764. The fragment ions (787.0900 Da and 769.0813Da) were caused by the loss of galloyl (152.0110 Da) and gallic acid (170.0197Da) moieties, respectively. Further loss of galloyl and gallic acid moieties revealed a fragmentation pattern typical of penta-*O*-galloyl-glucose. Compound 20 had a signal of molecular ion [M-H]^-^ at *m/z* 1091.1174(error, -3.57 ppm), which yielded fragment ions at *m/z* 939.0916 due to loss of two consecutive galloyl (152.0258 Da) moieties. Further loss of galloyl and gallic acid moieties revealed a fragmentation pattern typical of hexa-*O*-galloyl-glucoside. It was identified as hexa-*O*-galloyl-glucose.

4. Flavonoids

Among different compounds identified and detected in peel and pulp of mango cv. Ao, compounds such as 12, 13, 14, 15, 17, 18, 19, 21 and 22 were found to flavonoids. These flavonoids mainly were of different forms of glycosides. Among them quercetin compound was the major aglycone. Compounds 12 were identified and detected as quercetin 3-O-galactoside at *m/z* 463.0908[M-H]^-^ with MS^2^ fragment ions at *m/z* 301.0396 and 300.0330, which were similar with compounds 13, 14 and 17 typical of quercetin glycosides. Thus compounds 12, 13, 14 and 17 could unambiguously be identified were tentatively considered to be as quercetin 3-*O*-galactoside, quercetin 3-*O*-glucoside, iso-quercetin 3-*O*-glucoside and iso-quercetin 3-*O*-glucoside on the basis of the result reported by Berardini et al. [4]. Compounds 15 and 18 had a same signal isomers of molecular ion ([M-H]^-^) at *m/z* 433.0811with MS^2^ fragment ions at *m/z* 301.03, typical of quercetin glycosides, and possibly represent quercetin 3-*O*-xyloside and iso-quercetin 3-*O*-xyloside respectively, as described by[4]. Compound 19 was identified and detected as kaempferol 3-O-glucoside at *m/z* 447.0963[M-H]^-^ generating fragment ions at *m/z* 285.0455, 284.0391 and 225.0366. Compounds 21 and 22 exhibited a same molecular ion [M-H]^-^ at *m/z* 447.0960(error, 7.38 ppm) with MS^2^ fragment ions at *m/z* 300.0331and 300.0340. These two peaks were celebrated as quercetin 3-*O*-rhamnoside and iso-quercetin 3-*O*-rhamnoside, respectively, according the result reported by [4]. The phenolic compounds identified in pulp are quite similar to the compounds of peel. However, one compound in pulp was different from peel. This compound (t_R_ =9.783) was identified and detected as iriflophenone di-*O*-galloyl-glucoside at *m/z* 711.1124 ([M-H]^-^). It was regarded as 5 biomarkers, maclurin-mono-*O*-galloyl-glucoside (575.1030 Da), maclurin-di-*O*-galloyl-glucoside (727.1057 Da), maclurin tri-*O*-galloyl-glucoside (879.1256 Da), mangiferin gallate (573.088 Da), iriflophenone- di-*O*-galloyl-glucoside (711.1124 Da) for tolerance against *Fm* infection were identified in pulp.
